# Development and validation of an individualized diagnostic signature in thyroid cancer

**DOI:** 10.1002/cam4.1397

**Published:** 2018-03-09

**Authors:** Li‐ou Han, Xin‐yu Li, Ming‐ming Cao, Yan Cao, Li‐hong Zhou

**Affiliations:** ^1^ Department of Thyroid Surgery the First Affiliated Hospital of Harbin Medical University Harbin 150001 China; ^2^ Department of Endocrinology the First Affiliated Hospital of Harbin Medical University Harbin 150001 China

**Keywords:** Diagnostic, individualized, signature, thyroid cancer

## Abstract

New molecular signatures are needed to improve the diagnosis of thyroid cancer (TC) and avoid unnecessary surgeries. In this study, we aimed to develop a robust and individualized diagnostic signature in TC. Gene expression profiles of tumor and nontumor samples were from 13 microarray datasets of Gene Expression Omnibus (GEO) database and one RNA‐sequencing dataset of The Cancer Genome Atlas (TCGA). A total of 1246 samples were divided into a training set (*N *= 435), a test set (*N *= 247), and one independent validation set (*N *= 564). In the training set, 115 most frequent differentially expressed genes (DEGs) among the included datasets were used to construct 6555 gene pairs, and 19 significant pairs were detected to further construct the diagnostic signature by a penalized generalized linear model. The signature showed a good diagnostic ability for TC in the training set (area under receiver operating characteristic curve (AUC) = 0.976), test set (AUC = 0.960), and TCGA dataset (AUC = 0.979). Subgroup analyses showed consistent results when considering the type of nontumor samples and microarray platforms. When compared with two existing molecular signatures in the diagnosis of thyroid nodules, the signature (AUC = 0.933) also showed a higher diagnostic ability (AUC = 0.886 for a 7‐gene signature and AUC = 0.892 for a 10‐gene signature). In conclusion, our study developed and validated an individualized diagnostic signature in TC. Large‐scale prospective studies were needed to further validate its diagnostic ability.

## Introduction

Thyroid cancer (TC) is one of the most common cancers around the world 1. Although thyroid nodules were usually diagnosed as benign, 5~15% proved to be malignant 2, 3. Fine‐needle aspiration (FNA) biopsies were the common way to determine the malignancy of thyroid nodules. However, 15~30% of aspirations reported an indeterminate cytologic finding 4, 5. Most of these patients were recommended for a diagnostic thyroid surgery, but only 15~25% proved malignant 6. Thus, the surgery was unnecessary for a significant number of patients, which were exposed to a 2~10% risk of serious surgical complications. Moreover, lifetime levothyroxine supplementation and additional medical costs were also required 7. In clinical practice, it had a critical need to increase the diagnostic accuracy for TC.

Recently, several molecular signatures have been identified to improve TC diagnosis 8, 9. However, most signatures were limited in the sample size and absence of a specific diagnostic formula and cross‐validation. With the development of high‐throughput gene detection technology, gene expression profiles were available to identify more novel and robust biomarkers. On the other hand, diverse biological algorithms provided the possibility to integrate the samples in different batches and construct a more practical diagnostic or prognostic signature 10. In this study, we integrated a large number of samples and developed and validated an individualized diagnostic signature in TC.

## Method

### Data collection

Gene Expression Omnibus (GEO) database (http://www.ncbi.nlm.nih.gov/geo/) was searched for related gene expression profiles from inception to 1 October 2017. Datasets were included if fulfilled the following criteria: containing both TC samples and nontumor samples (adjacent normal tissues, thyroid adenoma, or healthy controls); based on the chip platform of Affymetrix Human Genome U133 Array (GPL96, GPL570, or GPL571); a sample size of more than 10. The datasets with a sample size of more than 50 and at least 20 nontumor samples were merged into one set for signature identification (training set), while the remaining were pooled into one set for signature validation (test set). Moreover, another RNA‐sequencing dataset from The Cancer Genome Atlas (TCGA) (https://cancergenome.nih.gov/) was also selected as an independent validation set.

### Data preprocessing

Normalized gene expression profiles were downloaded by an ftp method (http://ftp://ftp.ncbi.nlm.nih.gov/geo/series/). The probe IDs were matched to gene symbols using the Affymetrix annotation files (http://www.affymetrix.com). When multiple probes matched to an identical gene symbol, we took the average value of expression values across multiple probe IDs to represent the corresponded gene symbol 11.

### Differentially expressed genes (DEGs) screening

Linear model was used to screen the DEGs between tumor and nontumor samples in each included dataset of the training set. The false discovery rate (FDA) <0.05 and |log_2_ fold change (FC)| >0.585 were chosen as the cutoff criteria.

### Signature construction

In training set, the most frequent DEGs among the included datasets were selected, and the gene expression level in a specific sample underwent pairwise comparison with generate a score for each gene pair. If the first gene of a gene pair had a higher expression value than the second gene, a gene‐pair score of 1 was assigned; otherwise, the gene‐pair score was 0. Then, a LASSO penalized generalized linear model was used in the training set to identify significant gene pairs 12. The penalty parameter was estimated by 10‐fold cross‐validation at 1 standard error (SE) beyond the minimum partial likelihood deviance.

### Signature evaluation and validation

The coefficients of significant gene pairs in the model were extracted to calculate a diagnostic score for each sample in the training set, test set, and TCGA set. Receiver operating characteristic (ROC) curves and area under ROC curve (AUC) were used to evaluate the diagnostic ability of the signature score, validate it in different sets, and compare it with other molecular signatures.

### Statistical analysis

All statistical analyses were performed using R software (version 3.4.2, https://www.r-project.org/). DEG analysis was conducted with limma (version 3.6) package. The generalized linear model was constructed with glmnet package (version 2.0‐13). ROC analysis was performed with ROCR (version 1.0‐7) package. A two‐sided *P* value <0.05 was considered statistically significant.

## Results

### Characteristics of included datasets

Thirteen GEO datasets and one TCGA dataset were identified with a total of 1246 samples (925 tumor samples and 321 nontumor samples) (Table 1). Ten microarray datasets were based on the platform of GPL570, and three on GPL96. Five datasets (GSE27155, GSE33630, GSE35570, GSE60542, and GSE82208) were merged into a training set with a total of 263 tumor and 172 nontumor samples. The remaining eight datasets (GSE29265, GSE3467, GSE3678, GSE53157, GSE5364, GSE58545, GSE6004, and GSE65144) were pooled into a test set with a total of 157 tumor and 90 nontumor samples. Another independent validation set of TCGA had a total of 505 tumor and 59 nontumor samples.

**Table 1:** Details about the datasets used in this study

Accession	Year	Area	Platform	Number of samples		
				Total	Tumor	Nontumor
Training set						
GSE27155	2011	USA	GPL96	99	78	21
GSE33630	2012	Belgium	GPL570	105	60	45
GSE35570	2015	Poland	GPL570	116	65	51
GSE60542	2015	Belgium	GPL570	63	33	30
GSE82208	2017	Poland	GPL570	52	27	25
Test set						
GSE29265	2012	Belgium	GPL570	49	29	20
GSE3467	2005	USA	GPL570	18	9	9
GSE3678	2006	USA	GPL570	14	7	7
GSE53157	2013	Portugal	GPL570	27	24	3
GSE5364	2008	Singapore	GPL96	51	35	16
GSE58545	2015	Poland	GPL96	45	27	18
GSE6004	2006	USA	GPL570	18	14	4
GSE65144	2015	USA	GPL570	25	12	13
Independent validation set						
TCGA	2015	USA	IlluminaHiSeq	564	505	59

### Signature construction and evaluation

**Figure 1:**
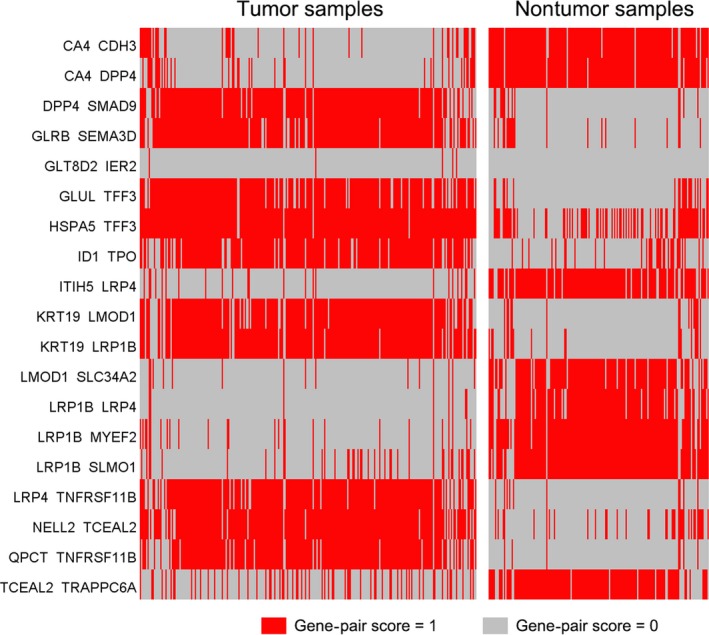
Heatmap of the gene‐pair scores in tumor and nontumor samples of training set.

**Figure 2:**
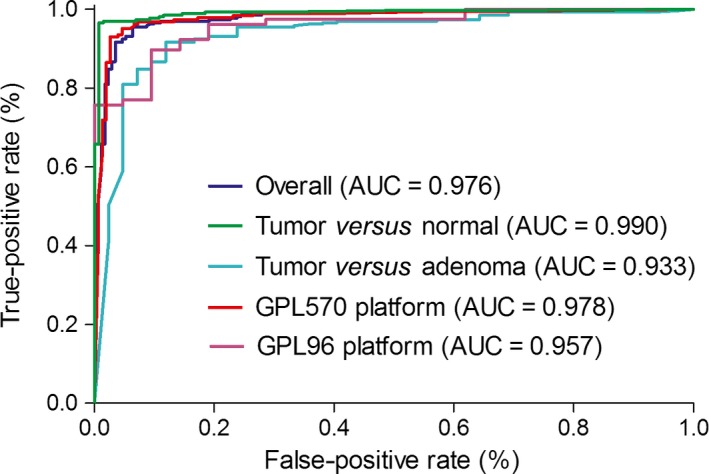
Receiver operating characteristic (ROC) curves and area under ROC curve (AUC) of the diagnostic signature in training set.

In training set, 115 genes were identified as DEGs among at least four included datasets (Figure S1). These genes were used to construct 6555 gene pairs. The LASSO penalized generalized linear model identified 19 significant gene pairs (consisting of 26 genes), which showed an obviously different distribution in tumor and nonsample samples (Fig. 1). Then, a diagnostic score was developed based on these gene pairs and their coefficients in the model (Table 2). The score showed a good diagnostic ability for TC (AUC = 0.976) (Fig. 2). Subgroup analyses also showed consistent results when considering the type of nontumor samples and microarray platforms.

**Table 2:** Signature information

Gene pair 1	Full name	Gene pair 2	Full name	Coefficient
CA4	Carbonic anhydrase IV	CDH3	Cadherin 3, type 1, P‐cadherin	−0.343957681
CA4	Carbonic anhydrase IV	DPP4	Dipeptidyl‐peptidase 4	−0.414152857
DPP4	Dipeptidyl‐peptidase 4	SMAD9	SMAD family member 9	0.198178763
GLRB	Glycine receptor, beta	SEMA3D	Sema domain, immunoglobulin domain (Ig), short basic domain, secreted, (semaphorin) 3D	0.213058352
GLT8D2	Glycosyltransferase 8 domain containing 2	IER2	Immediate early response 2	0.054809905
GLUL	Glutamate‐ammonia ligase	TFF3	Trefoil factor 3	0.106305040
HSPA5	Heat shock 70 kDa protein 5	TFF3	Trefoil factor 3	0.137164218
ID1	Inhibitor of DNA binding 1	TPO	Thyroid peroxidase	0.133836321
ITIH5	Interalpha‐trypsin inhibitor heavy chain family, member 5	LRP4	Low‐density lipoprotein receptor‐related protein 4	−0.449239654
KRT19	Keratin 19	LMOD1	Leiomodin 1	0.166685729
KRT19	Keratin 19	LRP1B	Low‐density lipoprotein receptor‐related protein 1B	0.026410689
LMOD1	Leiomodin 1	SLC34A2	Solute carrier family 34, member 2	−0.134480682
LRP1B	Low‐density lipoprotein receptor‐related protein 1B	LRP4	Low‐density lipoprotein receptor‐related protein 4	−0.180802140
LRP1B	Low‐density lipoprotein receptor‐related protein 1B	MYEF2	Myelin expression factor 2	−1.070962895
LRP1B	Low‐density lipoprotein receptor‐related protein 1B	SLMO1	Slowmo homolog 1	−0.158658033
LRP4	Low‐density lipoprotein receptor‐related protein 4	TNFRSF11B	Tumor necrosis factor receptor superfamily, member 11b	0.149105475
NELL2	NEL‐like 2	TCEAL2	Transcription elongation factor A (SII)‐like 2	0.178969566
QPCT	Glutaminyl‐peptide cyclotransferase	TNFRSF11B	Tumor necrosis factor receptor superfamily, member 11b	0.692483617
TCEAL2	Transcription elongation factor A (SII)‐like 2	TRAPPC6A	Trafficking protein particle complex 6A	−0.229715917

### Signature validation

**Figure 3:**
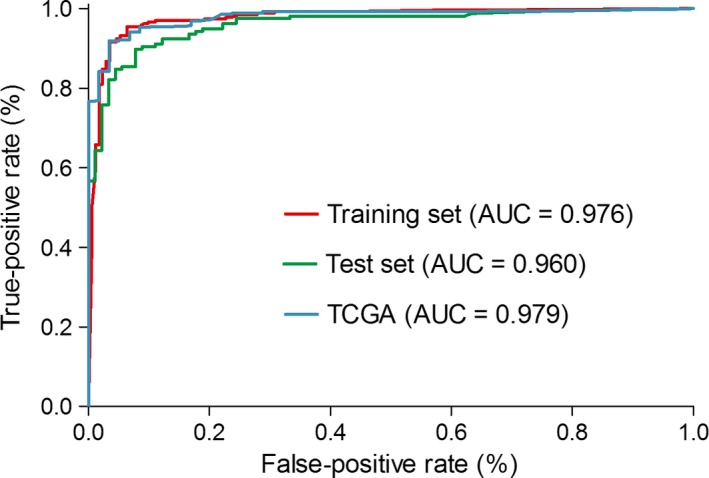
Receiver operating characteristic (ROC) curves and area under ROC curve (AUC) of the diagnostic signature in test set.

We also constructed the same 19 gene pairs in the test set and TCGA set. The coefficients of the 19 gene pairs in the model of training set were extracted to calculate a diagnostic score for each sample in test set and TCGA set. The score also showed a good diagnostic ability in both test set (AUC = 0.960) and TCGA set (AUC = 0.979) (Fig. 3).

### Comparison with other molecular signatures

**Figure 4:**
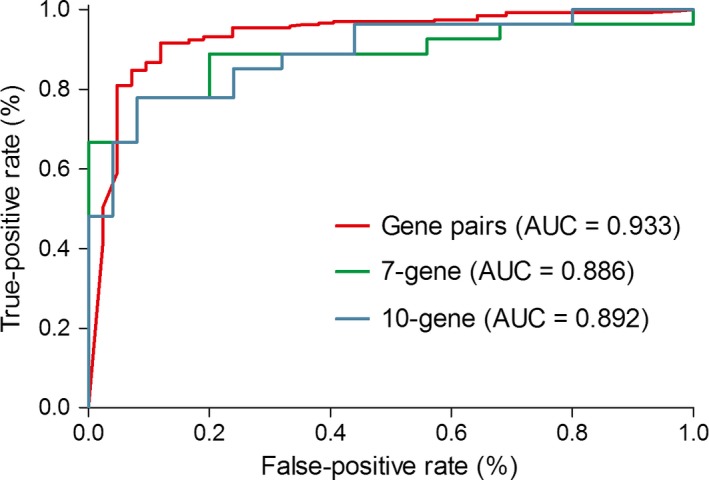
Receiver operating characteristic (ROC) curves and area under ROC curve (AUC) of different signatures in diagnosing thyroid nodules.

A 7‐gene signature and 10‐gene signature were published recently, both of which showed a good diagnostic ability for thyroid nodule malignancy 13, 14. As all nontumor samples in GSE82208 were obtained from thyroid nodules and diagnosed as follicular adenoma, this dataset was selected to reevaluate the two signatures using a logistics regression model. In result, the gene‐pair signature (AUC = 0.933) showed a better diagnostic ability than the 7‐gene signature (AUC = 0.886) and 10‐gene signature (AUC = 0.892) (Fig. 4).

## Discussion

With the development of microarray and RNA‐sequencing technology, we were entering a new era of big biological data. A tremendous amount of genomic information was detected in individual samples, which promoted the identification of novel biomarkers, therapeutic targets, and potential pathogenesis. However, most studies were limited in the sample size, and it was difficult to integrate the samples in different sets for batch effects. The TCGA plan has finished RNA sequencing of a relatively large number of tumor samples in multiple cancers. LncRNA and mRNA prognostic signatures of TC have been developed using the TCGA data 15, 16. However, few studies focused on the microarray and RNA‐sequencing data to identify diagnostic signatures in TC. The 167‐gene model by Alexander et al. was the first signature based on TC microarray data 17. It showed a good diagnostic ability, but it was really difficult for clinical practice, considering the lack of a specific diagnostic formula and cross‐validation based on large‐scale samples and multiple platforms.

Multiple microarray platforms occurred in recent years. Thereinto, Affymetrix Human Genome U133 Arrays were well illustrated and widely used. In this study, we reviewed all TC studies based on the Affymetrix U133 platform in GEO database and selected those with tumor and nontumor samples for further analyses. Datasets with a relatively large sample size were included for DEGs analysis. Considering the significant heterogeneity between studies, we chose the most frequent DEGs among individual studies as candidate genes. In gene ontology analysis, these DEGs showed an association with thyroid hormone metabolic process (Figure S2). Subsequently, we constructed 6555 gene pairs with the 115 DEGs. The signature consisting of 19 gene pairs showed a good diagnostic ability for TC. The 26 genes constituting the gene pairs also related with multiple biological processes, especially estrogen‐related process which was involved in the pathogenesis of TC 18, 19 (Figure S3). The gene‐pair signature reflected the expression imbalance of estrogen‐related genes, which could make significant effects on the development of TC.

This gene‐pair‐based method had an important advantage because the score was calculated based entirely on the gene expression profile of an individual sample and could be used in an individualized manner without the need for considering the batch effects 20, 21. By this method, we were able to integrate small‐scale studies into one large set, which increased the utilizing efficiency of public biological data. Furthermore, considering the heterogeneity between different gene detection technologies, we evaluated the robustness of our signature by a RNA‐sequencing dataset, and the signature had a stable performance. We also used the same data to compare the signature with other molecular signatures, and the signature showed a higher ability in diagnosing thyroid nodules.

In clinical application, it was very promising to develop a diagnostic kit which could measure the expression levels of 26 genes in the 19 gene pairs. Then, the sample could be diagnosed as malignant or benign according to the signature score and the threshold.

The limitations should be acknowledged for our study. First, this study was retrospective designed, although we tried to include as many datasets as possible and took a rigorous validation for our signature. Second, the gene‐pair method was an individualized method, but not all batch effects could be addressed, and some might remain.

In conclusion, our study developed and validated an individualized diagnostic signature in thyroid cancer. Large‐scale prospective studies were needed to further validate its diagnostic ability.

## Conflict of interest

None.

## Supporting information


**Figure S1.** Distribution of differentially expressed genes in five included datasets of the training set.**Figure S2.** Heatmap of enriched biological processes across the differentially expressed genes (colored by *P* values).**Figure S3.** Heatmap of enriched biological processes across the 26 genes in the signature (colored by *P* values).
