# Supplementary material for: Development and validation of an individualized diagnostic signature in thyroid cancer
Source: Cancer Med. 2018 Mar 9;7(4):1135–40. doi: 10.1002/cam4.1397 (PMC5911625; doi:10.1002/cam4.1397)
Supplement: Supplementary file 1 — Figure S1. Distribution of differentially expressed genes in five included datasets of the training set.Figure S2. Heatmap of enriched biological processes across the differentially expressed genes (colored by P values).Figure S3. Heatmap of enriched biological processes across the 26 genes in the signature (colored by P values). [file CAM4-7-1135-s001.doc]

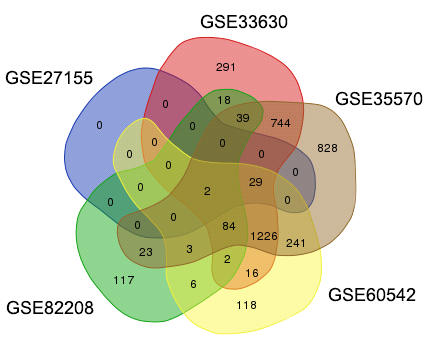


**Figure S1.** Distribution of differentially expressed genes in five included datasets of the training set.


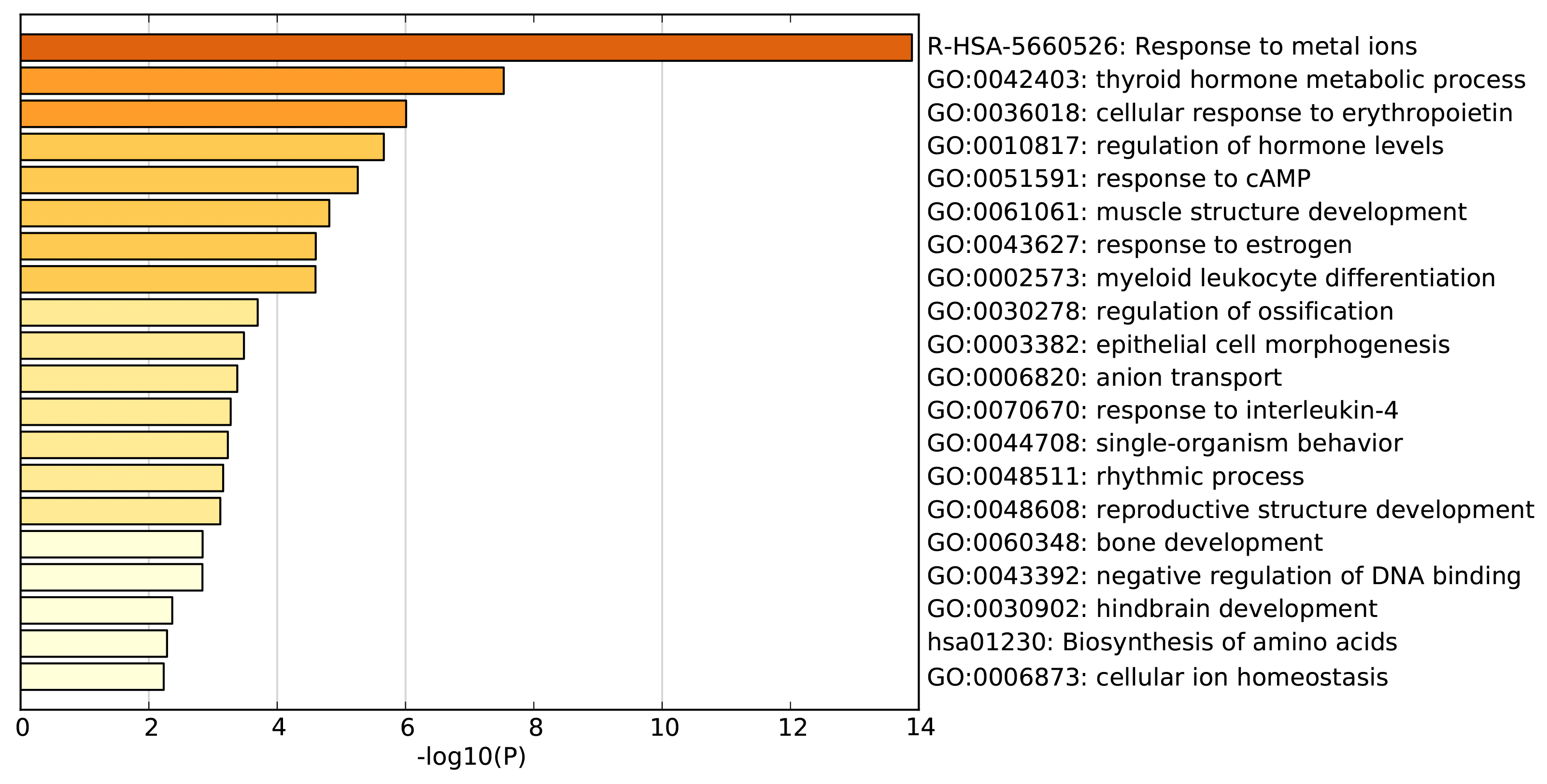


**Figure S2.** Heatmap of enriched biological processes across the differentially expressed genes (colored by *P* values).


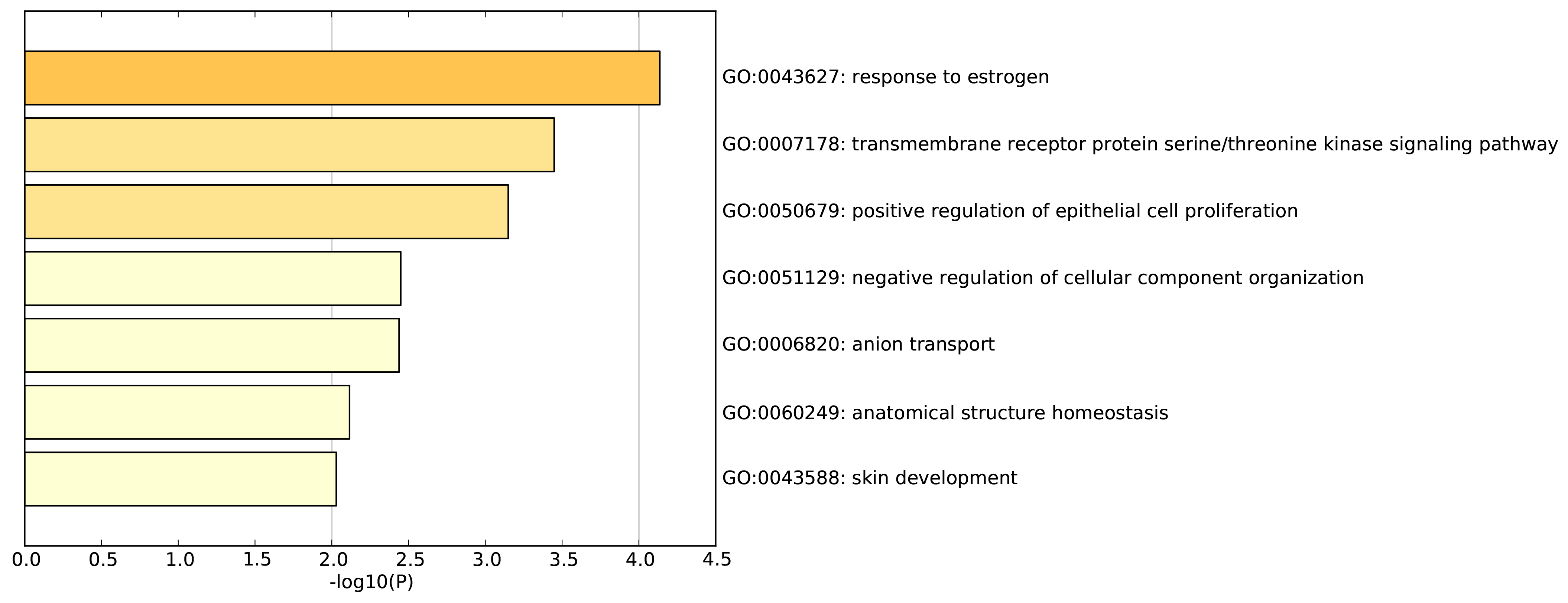


**Figure S3.** Heatmap of enriched biological processes across the 26 genes in the signature (colored by *P* values).
